# Retraction: Nontuberculous Mycobacterial Infection After Cholecystectomy: A Case Series and Literature Review

**DOI:** 10.7759/cureus.r260

**Published:** 2026-11-03

**Authors:** Chhavi Singh, Archana Khanduri, Alka R Chauhan, Rahul Gupta

**Affiliations:** 1 Microbiology, Synergy Institute of Medical Sciences, Dehradun, IND; 2 Gastrointestinal Surgery, Synergy Institute of Medical Sciences, Dehradun, IND

This article has been retracted by the Editor-in-Chief at the request of the corresponding author. A post-publication inquiry identified concerns about the accuracy of the reported clinical data and about patient consent for publication.

